# Context‐Dependent Movement Responses of Large Carabids to Forest Gap Cuttings

**DOI:** 10.1002/ece3.74250

**Published:** 2026-08-28

**Authors:** Jana Růžičková, Áron Székely, Péter Ódor, Zoltán Elek

**Affiliations:** ^1^ HUN‐REN‐ELTE‐MTM Integrative Ecology Research Group, Biological Institute ELTE Eötvös Loránd University Budapest Hungary; ^2^ Department of Systematic Zoology and Ecology, Biological Institute ELTE Eötvös Loránd University Budapest Hungary; ^3^ Institute of Ecology and Botany HUN‐REN Centre for Ecological Research Vácrátót Hungary; ^4^ Institute of Environmental Protection and Nature Conservation, Forestry Faculty University of Sopron Sopron Hungary; ^5^ Department of Biostatistics University of Veterinary Medicine Budapest Budapest Hungary; ^6^ HUN‐REN‐DE Anthropocene Ecology Research Group University of Debrecen Debrecen Hungary

**Keywords:** continuous cover forestry, hidden Markov models, insect behavior, radio‐tracking, telemetry

## Abstract

Continuous cover forestry aims to mimic natural disturbance dynamics and thus preserve the structure of uneven‐aged, semi‐natural forests. A common practice within this silvicultural system is the selective logging of single trees or small groups of trees, creating small‐scale canopy gaps that can modify local environmental conditions and potentially affect the movement of ground‐dwelling insects. Here, we examined movement responses of two large, flightless carabid beetles, *Carabus coriaceus* and *C. ullrichii* (Coleoptera: Carabidae), in a managed oak‐hornbeam forest in Hungary. Using radio telemetry, we tracked 27 individuals at 8‐h intervals for up to ten days following release in either gap cuttings or adjacent closed‐canopy forest. Individual movement trajectories were quantified using daily dispersal, net displacement, and hidden Markov models to distinguish random walks (a proxy for foraging) from directed movements (dispersal). The two species showed contrasting movement patterns. *Carabus ullrichii* had shorter step lengths and occupied smaller spatial areas, with lower daily dispersal in gap cuttings than in closed‐canopy forest and only marginal sex effects. In contrast, the movement of *C. coriaceus* was unaffected by gap cuttings, suggesting limited sensitivity to this habitat type, but strongly sex‐dependent, with males covering larger daily and net distances and showing a higher tendency for directed movement than females. The proportion of random walks did not vary with habitat in either species. Our results demonstrate that even small‐scale forestry interventions can trigger highly species‐ and sex‐specific movement responses, revealing behavioral shifts that remain entirely undetected by traditional, assemblage‐level sampling approaches such as pitfall trapping.

## Graphical abstract

This study investigates the effects of forest management on the movement of ground beetles within an oak‐hornbeam forest in northern Hungary. We compared a closed‐canopy control stand with various small‐scale canopy gaps. To track movement and habitat use within these treatments, two carabid species, *Carabus ullrichii* and *C. coriaceus* , were followed using radio telemetry.

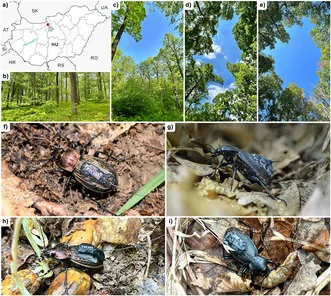

## 1 Introduction

In European temperate forests, spontaneous canopy gaps represent the main form of natural disturbance. These gaps are created when one or a few canopy trees die, are damaged, or lose large branches (Yamamoto 2000; Standovár and Kenderes 2003). As they are subsequently filled by regenerating trees, gap dynamics maintain characteristic structural and spatial forest heterogeneity (Schliemann and Bockheim 2011). However, only a small fraction of European forests (around 2%; Forest Europe 2020) can be considered natural and unaffected by human activity, while most stands are currently managed under various, predominantly large‐scale forestry systems (Bengtsson et al. 2000; Paillet et al. 2010). In response, there is a growing effort to implement management approaches that enhance structural complexity within and between forest stands and better align timber production with natural disturbance regimes (Burrascano et al. 2023; Müller et al. 2023; Pierick, Link, et al. 2026). One widely discussed alternative to rotational large‐scale clear‐cutting is Continuous Cover Forestry (CCF; Pommerening and Murphy 2004). However, traditional CCF relies primarily on single‐tree selection and thinning, which maintains a continuously closed canopy (Helliwell 1997) rather than fully mimicking the natural gap dynamics with early‐successional stages essential for forest regeneration and biodiversity (Brunet et al. 2010; Hilmers et al. 2018). To incorporate these missing dynamics, other management options within CCF can include group selection or irregular shelterwood systems. Among these, gap cutting is one of the most common approaches, creating small canopy openings that mimic natural tree falls (Schliemann and Bockheim 2011; Mason et al. 2022).

Despite their relatively small size, often less than 0.1 ha in temperate forests (Zhu et al. 2015), gap cuttings can still affect environmental conditions, including microclimate, vegetation structure, and biodiversity, although their impacts are generally less pronounced than those of large‐scale clear‐cutting (Kovács et al. 2020; Lettenmaier et al. 2025; Rothacher et al. 2025; Pierick, Seidel, et al. 2026). In gap cuttings with a 1:1 gap‐diameter‐to‐tree‐height ratio, soil moisture is typically higher, primarily driven by reduced tree transpiration and canopy interception, along with increased solar radiation and higher average daily air temperatures compared to the surrounding closed‐canopy forest (Gálhidy et al. 2006; Bagnato et al. 2021; Horváth et al. 2023). However, at the forest floor, soil and ground‐level temperatures remain largely buffered because elevated soil moisture supports evaporative cooling (Horváth et al. 2023), allowing the regeneration of shade‐intolerant tree species and increasing ground vegetation density (Schliemann and Bockheim 2011; Tinya et al. 2025, 2026).

This fine‐scale environmental heterogeneity in gap cuttings may be particularly relevant for various forest arthropods, whose small body size and close association with the forest floor, leaf litter, or dead wood make them sensitive to even subtle habitat alterations (Goßner et al. 2006; Sebek et al. 2015; Perry et al. 2018; Samu et al. 2023; Decker et al. 2026). Among them, carabid beetles (Coleoptera: Carabidae) are an abundant, diverse, and ecologically important group of ground‐dwellers that often serve as bioindicators of environmental change, particularly in relation to local habitat conditions, management, hydrological shifts, and drought stress in forests (Jambrošić Vladić and Šerić Jelaska 2020; Ludwiczak et al. 2020; Wang et al. 2021; Weiss et al. 2024). A key caveat, however, is that carabid responses can be highly context‐dependent.

Gap cuttings are typically associated with only minor changes in carabid assemblage composition, abundance, species richness, and functional diversity, resulting in assemblages resembling those found in semi‐natural forests with a closed canopy (Koivula and Niemelä 2002; Ulyshen et al. 2006; Elek et al. 2022). However, responses of carabids to gap cuttings may become more apparent at lower levels of organization. At the species level, carabid species sharing similar functional traits, for instance, large flightless forest specialists, can still show contrasting numerical responses to gap cuttings when certain species disappear while others remain relatively unchanged in terms of their abundance (Růžičková et al. 2025). At the behavioral level, individuals may adjust their movement activity in response to local habitat conditions. For instance, increased activity can reflect an immediate effort to seek out more suitable habitat patches, making altered movement patterns valuable early indicators of environmental change (Brouwers and Newton 2009). Given that most studies on carabids in managed forests are based on traditional pitfall trapping and assemblage‐level responses, focusing on individual movement has strong potential to complement them and reveal immediate behavioral mechanisms behind patterns detected at the species and assemblage levels (Růžičková and Elek 2026).

In temperate forests, the individual movement of large carabids has been documented regarding general habitat use (Rijnsdorp 1980; Riecken and Raths 1996; Negro et al. 2008; Kagawa and Maeto 2009; Růžičková and Veselý 2018) and large‐scale forestry treatments (Skłodowski 2008; Negro et al. 2017; Elek et al. 2021), yet their behavioral responses to gap cuttings within CCF remain poorly understood. In this study, we therefore focused on the movement patterns of two large and common forest species, *Carabus ullrichii* Germar, 1824, and *C. coriaceus* L., 1758, that can be frequently found in managed oak‐hornbeam forests in Hungary (Andorkó and Kádár 2006; Kádár et al. 2015; Fülöp et al. 2021; Elek et al. 2022) and have not shown a prominent decline in recent years, unlike some other previously common forest carabid species (Růžičková et al. 2025). Despite their classification as forest generalists (Thiele 1977; Turin et al. 2003), both *C. coriaceus* and *C. ullrichii* have exhibited increases in abundance immediately following large‐scale forestry interventions, including clear‐cuttings (Růžičková et al. 2025). Previous studies suggest that these species may utilize clear‐cuttings and their associated edges for foraging or mating, possibly to mitigate competition with other large beetles of the genus *Carabus* in closed‐canopy forests (Růžičková and Veselý 2018; Elek et al. 2021; Růžičková et al. 2021). However, exploring these open areas likely involves a trade‐off, as individuals may face a higher risk of predation (Růžičková and Elek 2021a).

Here, we examined the movement responses of these two species to gap cuttings (hereinafter referred to as gaps) of various shapes, created within the CCF experimental framework in a Hungarian oak‐hornbeam forest. Based on the above‐mentioned clues, we presumed that movement responses in these fine‐scale gaps would be less pronounced than large‐scale clear‐cuttings (Elek et al. 2021); however, we expected at least some detectable shifts in behavior driven by differences in soil moisture, light intensity, temperature variability, and vegetation structure in gap cuttings compared to adjacent closed‐canopy forest (Horváth et al. 2023; Tinya et al. 2025). Moreover, these shifts could be further modified by the distinct morphology and phenology of the two species. While the smaller *C. ullrichii* typically reproduces in spring with summer larvae, the larger *C. coriaceus* reproduces in autumn and develops predominantly biennially (Hůrka 1996; Turin et al. 2003). To reveal underlying behavioral mechanisms, we used very high frequency (VHF) radio telemetry, an ideal tool for tracking the fine‐scale movement of large beetles and other insects under field conditions (Kissling et al. 2014; Růžičková and Elek 2023). By analyzing movement metrics, specifically daily dispersal, net displacement, and the proportion of two behavioral states within the trajectory (so‐called *random walks* and *directed movements*, Baars 1979; Rijnsdorp 1980), we can assess how individuals utilize specific habitat patches. Specifically, we tested three primary questions:

1. *Habitat recognition*: Do movement trajectories indicate that carabids perceive gaps as distinct habitats (e.g., localized random walks with shorter step lengths and higher turning angles indicating habitat preference)?
2. *Gap geometry*: Does gap shape (circular vs. elongated) alter the structural characteristics of their movement trajectories?
3. *Species‐specificity*: Are these movement responses species‐ and sex‐specific, reflecting the differing morphology (body size) and phenology (spring or autumn breeding season) of the two focal species?

## 2 Material and Methods

### 2.1 Study Site and Experimental Design

**FIGURE 1:**
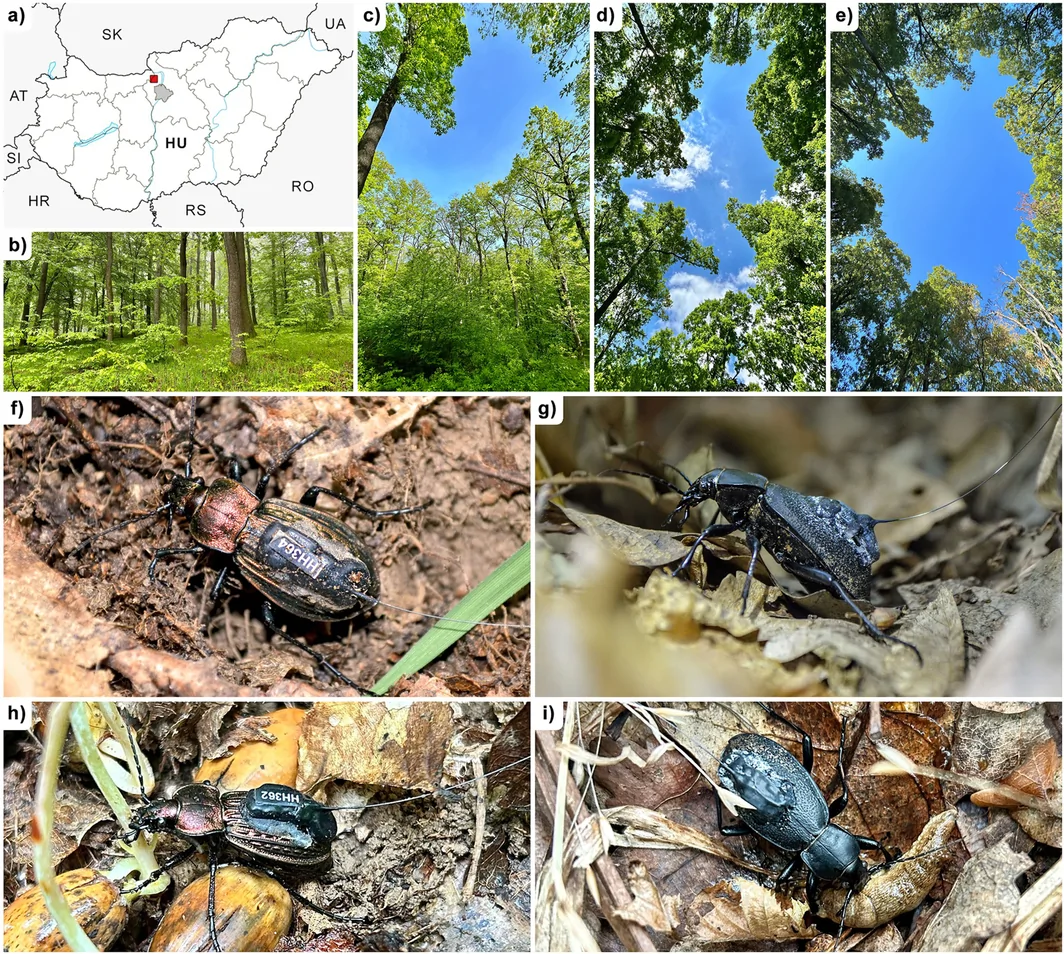
Location of the study area (Pilis Gap Experiment) in the Pilis Mountains, northern Hungary (a); closed‐canopy oak‐hornbeam forest serving as the control stand with a developed herb layer (b); artificial canopy gap with spontaneous tree regeneration in its center (c); large elongated (d) and large circular (e) canopy openings. *Carabus ullrichii* (f, h; body size: 22–33 mm) and *C. coriaceus* (g, i; body size: 33–40 mm) equipped with small radio transmitters; the male shown in (i) is preying on a slug.

The study area was located on the northern slope of Hosszú‐hegy (480 m a.s.l.) in the Pilis Mountains, near the village of Pilisszántó, northern Hungary (47.67048° N, 18.91609° E, Figure 1a). The closed‐canopy forest stand was an approximately 90‐year‐old oak‐hornbeam forest with a relatively uniform structure and homogeneous tree species composition, due to the formerly applied rotation forestry (shelterwood cutting). The stand was dominated by sessile oak ( *Quercus petraea* (Matt.) Liebl.), with occasional occurrence of turkey oak ( *Q. cerris* L.) in the upper canopy and hornbeam ( *Carpinus betulus* L.) and manna ash ( *Fraxinus ornus* L.) forming the lower canopy layer. While the shrub layer was sparse, with sporadic aggregated patches of hornbeam and manna ash regeneration, the herb layer was dense, composed of general and mesic forest species, such as *Carex pilosa* Scop. and *Melica uniflora* Retz. (Figure 1b). The site was part of the Pilis Gap Experiment (project website: https://piliskiserlet.ecolres.hu/en), where four gap treatments under the CCF framework were established by foresters from Pilisi Parkerdő Ltd. in the winter of 2018/2019 following a randomized complete block design. These artificial gaps of different sizes (small and large) and shapes (circular and elongated) were created by cutting and removing trees, which favored spontaneous tree regeneration (Figure 1c).

In this study, we specifically focused on two large gap treatments alongside a closed‐canopy control: (i) a large elongated gap measuring 30 m × 10 m (length × width; area ~ 300 m^2^) oriented north–south (Figure 1d); (ii) a large circular gap 20 m in diameter (1:1 gap‐diameter‐to‐tree‐height ratio; area ~ 300 m^2^; Figure 1e); and (iii) a control in the surrounding closed‐canopy forest (Figure 1b). We selected these large gaps because their size seems to be sufficient to be perceived as distinct habitat patches by large carabid beetles, given their movement activity in forests (Riecken and Raths 1996; Růžičková and Veselý 2018; Elek et al. 2021). Furthermore, large gaps exhibit the strongest microclimatic contrast with the closed‐canopy forest interior, characterized by higher soil moisture, greater solar radiation, and increased temperature variability (Horváth et al. 2023). These gaps also already had a well‐developed oak and hornbeam regeneration layer in their centers (Tinya et al. 2026, Figure 1c). However, as preliminary analyses revealed no differences in beetle movement between elongated and circular gaps, these two gap shapes were merged into a single “gap” treatment category (see Section 2.3).

### 2.2 Sampling and Radio‐Tracking of Ground Beetles

We selected two large carabid species that are common forest carabids in the study area, *C. ullrichii* and *C. coriaceus* . Living individuals were collected at the study site using pitfall traps partially filled with moss and leaves to prevent desiccation, with a few drops of beer used as an attractant. Beetles were collected during their respective breeding seasons: *C. ullrichii* between late April and early May, and *C. coriaceus* in September. Captured beetles were kept indoors with natural lighting conditions in large plastic containers filled with moss, leaf litter, and dead wood, with sexes separated. The sex of the beetles was determined based on the distinctive shape of their front tarsi. Beetles were fed ad libitum every two days with wet cat food and white grapes. One day before the tracking experiment, each individual was weighed to one decimal place, and the elytra were cleaned with a damp cloth containing a drop of detergent to remove grease. Small VHF radio transmitters (Lotek PicoPip, type Ag337; weight 0.32 g; frequency range 150.001–150.999 MHz; size 13 × 5 × 3 mm; Lotek Ltd., Wareham, UK) with a 5 cm antenna oriented backwards were attached to the elytra using gel superglue (Loctite Superbond Powerflex Gel, Henkel Ltd., Düsseldorf, Germany) mixed with baking soda to ensure strong adhesion and a watertight seal. Each beetle was then kept individually without food in a small plastic container until the glue had fully cured, that is, until release the next morning. In total, eight individuals of *C. ullrichii* (four males and four females, Figure 1f,h) and 20 individuals of *C. coriaceus* (10 males and 10 females, Figure 1g,i) were equipped with radio transmitters.

Beetles were released in the morning hours either in a closed‐canopy forest serving as the control stand or in gap cuttings, and the latitude and longitude coordinates of their release locations (initial fixes) were recorded using a handheld GPS device (Garmin Dakota 20; WGS84 coordinate reference system; EPSG: 4326). Four individuals (two males and two females) of *C. ullrichii* were released in gaps and four in the adjacent closed‐canopy forest. For *C. coriaceus* , eight individuals (four males and four females) were released in gaps and 12 in the closed‐canopy forest. Individuals were tracked at 8‐h intervals for up to 10 days or until signal loss (e.g., due to predation), using AR8000 (AOR Ltd., Tokyo, Japan) and Sika (Lotek Ltd., Wareham, UK) handheld receivers with a directional Yagi antenna and a 20 cm dipole antenna for short‐range detection.

A homing procedure was used for tracking, whereby the search for a tagged beetle started at its last known position (the previous fix marked with a numbered bamboo stick) and followed the transmitter signal to within approximately 0.5 m to avoid crushing the beetle underfoot. A new bamboo stick was then installed at the updated location. Because fine‐scale GPS error can lead to overestimation of movement distances and incorrect turning angles (Růžičková and Elek 2021b), new fixes were recorded using a distance‐bearing approach with a measuring tape and the built‐in Compass application on an iPhone 14 (version iOS 18), corrected for magnetic declination. Movements shorter than 0.5 m were classified as passive fixes, indicating no detectable activity. *C. ullrichii* was tracked between 6 and 16 May 2025, whereas *C. coriaceus* was tracked between 1 and 9 October 2025 to cover the main activity period of the two species, respectively. After the experiment ended, the still‐living individuals were found, their transmitters were removed on site, and the beetles were immediately released. One male of *C. ullrichii* was predated on the second day after release (beetle ID: u01, body mass of 0.9 g, signal lost on 8 May 2025) and, because it had only one active fix, it was excluded from further analyses.

### 2.3 Data Analysis

From measured step lengths (distance covered between tracking sessions, i.e., in 8‐h intervals) and turning angles (azimuths), we calculated basic movement metrics for each individual, including total path length (the sum of all step lengths), net displacement (the straight‐line distance between the initial and last fix), and daily dispersal (the distance traveled by an individual within 24 h), along with associated standard errors for mean estimates. These metrics follow minimum reporting standards for ground‐dwelling insect movement ecology (Růžičková and Elek 2026) and characterize how individuals move through space, providing a basis for linking individual behavioral decisions to external factors such as habitat use.

Movement trajectories were visualized in QGIS 3.34 Prizren (QGIS Development Team 2023). Latitude and longitude coordinates of release locations were converted to the Universal Transverse Mercator coordinate system (UTM, Zone 34N; EPSG: 32634) using an online coordinate converter (https://epsg.io/transform, accessed 7 January 2026). This planar projection represents positions on a flat, two‐dimensional grid and provides greater positional precision for fine‐scale insect movement trajectories than geographic coordinate systems (Růžičková and Elek 2026). The Azimuth and Distance plugin in QGIS (ver. 0.9.19; De Paulo et al. 2024) was then used to reconstruct individual trajectories by sequentially projecting each fix from the initial location based on the measured step lengths (in meters) and azimuths (in degrees), from which UTM coordinates for all fixes were then derived.

All subsequent analyses were performed in R version 4.3.2 (R Core Team 2023). To identify species‐specific movement states, we fitted hidden Markov models (HMMs) to individual trajectories separately for each species using the *fitHMM* function from the “moveHMM” package (Michelot et al. 2016). We specified two latent behavioral states, reflecting well‐known movement patterns in carabids: a *random walk*, characterized by short steps and frequent turns, which serves as a proxy for foraging, and *directed movement*, characterized by long steps and small turning angles, which can be interpreted as dispersal or migration away from unfavorable sites (Baars 1979; Rijnsdorp 1980). A higher proportion of random walks within a trajectory indicates area‐restricted movement, suggesting habitat preference. Because HMMs can be complex and converge to local rather than global optima, model fitting is sensitive to the choice of initial parameter values. To reduce the risk of convergence to suboptimal solutions, we applied a multi‐start optimization approach as recommended by Michelot and Langrock (2025). Separately for each species, we fitted 1000 candidate models using randomly generated initial parameter values drawn from biologically meaningful ranges based on the previous movement studies on large carabids in forests (Růžičková and Veselý 2018; Elek et al. 2021). Initial values for mean step lengths and standard deviations were drawn from uniform distributions as 0.5–5 m for random walks and 2–30 m for directed movements. The zero‐mass parameter, representing the proportion of passive fixes, was drawn uniformly between 0.1 and 0.9 for both states. Turning angles were modeled with von Mises distributions, with mean angles fixed at π for random walks and 0 for directed movements, and concentration parameters were set to 0.5–2 for random walks and 5–20 for directed movements. Each candidate model was fitted independently, and model convergence was assessed based on the negative log‐likelihood. The final model for each species was selected as the one with the lowest negative log‐likelihood among all candidate fits (see Figure S1). The best models were then used to estimate state‐dependent movement parameters and to visualize trajectories decoded into two behavioral states using the Viterbi algorithm, with fixes colored by their inferred state.

To test for sex‐ and treatment‐specific differences in movement behavior, we fitted species‐specific generalized linear models using the *glmmTMB* function from the “glmmTMB” package (Brooks et al. 2017). Three movement responses were analyzed: (i) daily dispersal, representing the mean distance moved within 24 h; (ii) net displacement, defined as the Euclidean distance between the first and final fixes; and (iii) the proportion of random walk, calculated as the relative frequency of fixes classified as random walk based on the HMMs and fitted as a two‐column response using the *cbind* function. Daily dispersal and net displacement were modeled using a Gamma distribution with a log link, whereas the proportion of random walk was analyzed using a beta‐binomial distribution with a logit link. In all model types, sex and treatment (gap vs. closed‐canopy forest) were included as fixed effects. Models were initially fitted with a sex × treatment interaction; as this interaction was not significant in any model, it was removed, and only main effects were retained in the final models. Preliminary analysis further showed no differences between circular and elongated gaps for any response variable, thus not supporting our second research question about beetle sensitivity to gap geometry. These gap types were therefore merged into a single “gap” treatment to improve statistical power and robustness. Model fit was assessed by simulating residuals using the *simulateResiduals* function from the “DHARMa” package to detect potential violations of model assumptions (Hartig et al. 2024, also see Figure S2). The significance of fixed effects was evaluated using ANOVA with Type III estimates for the sum of squares using the “car” package (Fox and Weisberg 2018). Model‐predicted means (estimated marginal means) and corresponding confidence intervals for the studied fixed effects were obtained using the *emmeans* function from the “emmeans” package (Lenth 2025).

## 3 Results

We obtained movement data from 27 individuals of two species. Out of 666 collected fixes, 223 (33.5%) were active, that is, with any detectable movement longer than 50 cm, while 443 (66.5%) showed no movement. Mean step length over the 8‐h interval was 1.1 m for *C. ullrichii* (range: 0.5–6.5 m) and 4.2 m for *C. coriaceus* (0.5–37.6 m). Total distance covered during the tracking period varied notably among individuals, irrespective of species or sex. On average, *C. ullrichii* individuals covered 12.7 m (3.4–28.0 m), whereas *C. coriaceus* covered 29.7 m (1.8–181 m). Additional basic movement metrics for each tracked individual are summarized in Table 1.

**TABLE 1:** Summary of individual movement metrics for radio‐tracked beetles.

ID	Species	Sex	Treatment	Body mass (g)	Load (%)	Total distance (m)	Net displacement (m)	Average daily dispersal (m) ± SEM	No. of fixes	Active fixes
u03	*C. ullrichii*	Female	Gap	1.1	27.27	9.5	1.39	0.95 ± 0.182	30	14
u04	*C. ullrichii*	Male	Gap	0.8	37.50	7.3	2.44	0.76 ± 0.271	27	9
u05	*C. ullrichii*	Female	Gap	1.1	27.27	3.4	1.21	0.49 ± 0.139	19	5
u06	*C. ullrichii*	Male	Control	1.0	30.00	8.3	3.44	0.83 ± 0.505	30	7
u07	*C. ullrichii*	Female	Control	1.1	27.27	28.0	10.64	3.11 ± 1.210	26	16
u08	*C. ullrichii*	Male	Control	0.8	37.50	12.4	7.52	1.38 ± 0.407	25	13
u09	*C. ullrichii*	Female	Control	1.4	21.43	20.0	2.21	2.00 ± 0.682	30	17
c01	*C. coriaceus*	Female	Gap	2.6	11.54	2.2	0.37	0.28 ± 0.128	25	3
c02	*C. coriaceus*	Male	Gap	1.7	17.65	53.3	20.02	6.66 ± 2.248	25	16
c03	*C. coriaceus*	Female	Gap	2.5	12.00	2.5	0.52	0.42 ± 0.254	18	3
c04	*C. coriaceus*	Male	Gap	1.8	16.67	23.7	15.33	2.96 ± 1.617	25	12
c05	*C. coriaceus*	Female	Gap	2.7	11.11	20.0	16.19	2.50 ± 1.663	25	5
c06	*C. coriaceus*	Male	Gap	1.9	15.79	20.2	11.07	2.53 ± 1.014	25	10
c07	*C. coriaceus*	Female	Gap	2.5	12.00	14.0	10.54	1.75 ± 1.055	25	6
c08	*C. coriaceus*	Male	Gap	1.6	18.75	29.7	16.85	3.71 ± 1.539	25	12
c09	*C. coriaceus*	Female	Control	2.7	11.11	5.7	1.80	0.71 ± 0.220	25	6
c10	*C. coriaceus*	Male	Control	1.6	18.75	79.8	47.42	9.98 ± 3.648	25	12
c11	*C. coriaceus*	Female	Control	2.7	11.11	29.4	22.13	3.68 ± 2.517	25	6
c12	*C. coriaceus*	Male	Control	1.7	17.65	43.3	38.80	5.41 ± 4.315	25	6
c13	*C. coriaceus*	Female	Control	1.7	17.65	3.2	3.09	0.80 ± 0.693	11	2
c14	*C. coriaceus*	Male	Control	1.5	20.00	32.5	29.39	4.06 ± 3.078	25	3
c15	*C. coriaceus*	Female	Control	2.5	12.00	9.7	2.74	1.21 ± 0.514	25	7
c16	*C. coriaceus*	Male	Control	1.7	17.65	28.8	23.32	3.60 ± 1.671	25	7
c17	*C. coriaceus*	Female	Control	2.3	13.04	10.8	6.13	1.35 ± 1.099	25	3
c18	*C. coriaceus*	Male	Control	1.7	17.65	181.0	56.50	22.63 ± 5.255	25	17
c19	*C. coriaceus*	Female	Control	2.3	13.04	1.8	0.38	0.23 ± 0.104	25	3
c20	*C. coriaceus*	Male	Control	1.6	18.75	3.3	1.10	0.41 ± 0.253	25	3

*Note:* For each individual, we report species, sex, and treatment (habitat type), along with body mass (g) and transmitter load (% of body mass). Movement metrics include total distance traveled, net displacement between the first and last fix, and daily dispersal with standard error as a proxy for overall activity and space use. The total number of fixes and the number of active fixes indicate sampling effort and time spent moving.

**FIGURE 2:**
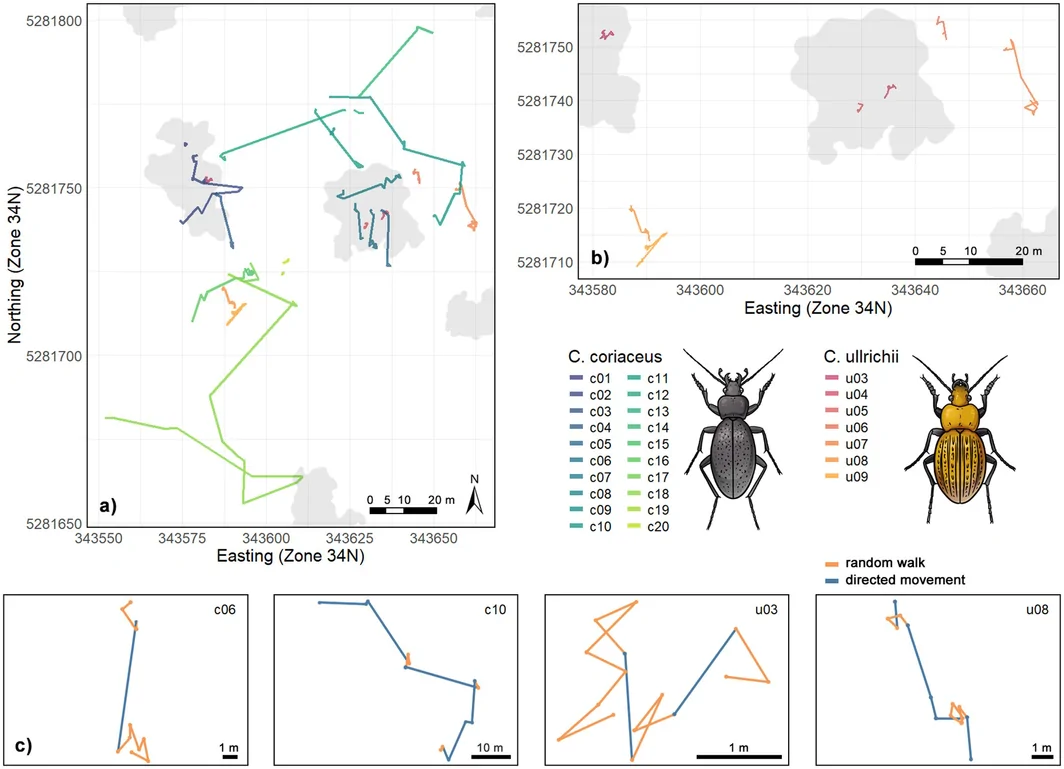
Movement trajectories of two large carabid species in a gap‐forest mosaic. Trajectory maps show movements of both *Carabus ullrichii* and *C. coriaceus* (a, geographic coordinates for the release point of beetle “c01” are 47° 40′ 13.2″ N, 18° 54′ 58.4″ E), and fine‐scale paths of *C. ullrichii* in greater detail (b). Gray silhouettes indicate the artificial canopy gaps. Four selected individual movement paths are shown separately, with the beetle ID shown in the upper right corner (c). These trajectories were decoded into two distinct behavioral states: orange segments represent random walk and blue segments are directed movement.

Consistent with these differences, trajectory maps (Figure 2a,b) showed that *C. ullrichii* moved within a much smaller spatial area than *C. coriaceus* . Moreover, individuals of *C. ullrichii* that were released in gap cuttings remained within this type of habitat and did not enter the surrounding closed‐canopy forest, and vice versa. Some of the gap‐released individuals of *C. coriaceus* occasionally reached the gap edge and moved along its perimeter, while some forest‐dwelling individuals (mostly males) were able to overcome a distance greater than the diameter of the canopy gap between tracking sessions. On the contrary, some other beetles remained inactive for multiple consecutive days while hidden in the leaf litter or dead wood. Smaller *C. ullrichii* usually moved within a thick layer of fallen leaves, whereas the larger *C. coriaceus* walked mostly on the surface of the forest floor and was frequently observed during tracking sessions. In one case, a tracked beetle (male) was observed feeding on a slug (Figure 1i).

HMMs with two latent behavioral states effectively captured movement patterns in both species, distinguishing random walks from directed movements (see examples of segmented trajectories in Figure 2c). In *C. ullrichii* (log‐likelihood = −244.24), random walks consisted of short steps (0.60 ± 0.12 m, mean ± SD) with frequent directional changes (mean turning angle = 2.88 rad, concentration parameter *κ* = 1.28) and negligible zero‐mass, whereas directed movement had longer steps (1.90 ± 1.29 m), more persistent orientation (mean turning angle = 0.22 rad, *κ* = 0.74), and a high zero‐mass component (0.77). *C. coriaceus* (log‐likelihood = −674.91) showed a similar pattern but on a larger scale: random walks showed short steps (1.12 ± 0.47 m, mean turning angle = 2.71 rad, *κ* = 1.05) with pronounced zero‐mass (0.81), while directed movement had much longer and more variable steps (8.07 ± 8.08 m) with a highly oriented direction (mean turning angle = 0.07 rad, *κ* = 0.45) and minimal zero‐mass (0.03). State‐switching dynamics were asymmetric in both species: *C. ullrichii* had higher transition probability to switch from random walk to directed movement (0.62) than the reverse (0.24), with higher persistence of directed movements (0.76), whereas *C. coriaceus* showed high persistence of random walks (0.95) and a lower transition probability to directed movement (0.04), with the opposite transition probability being higher (0.32).

**FIGURE 3:**
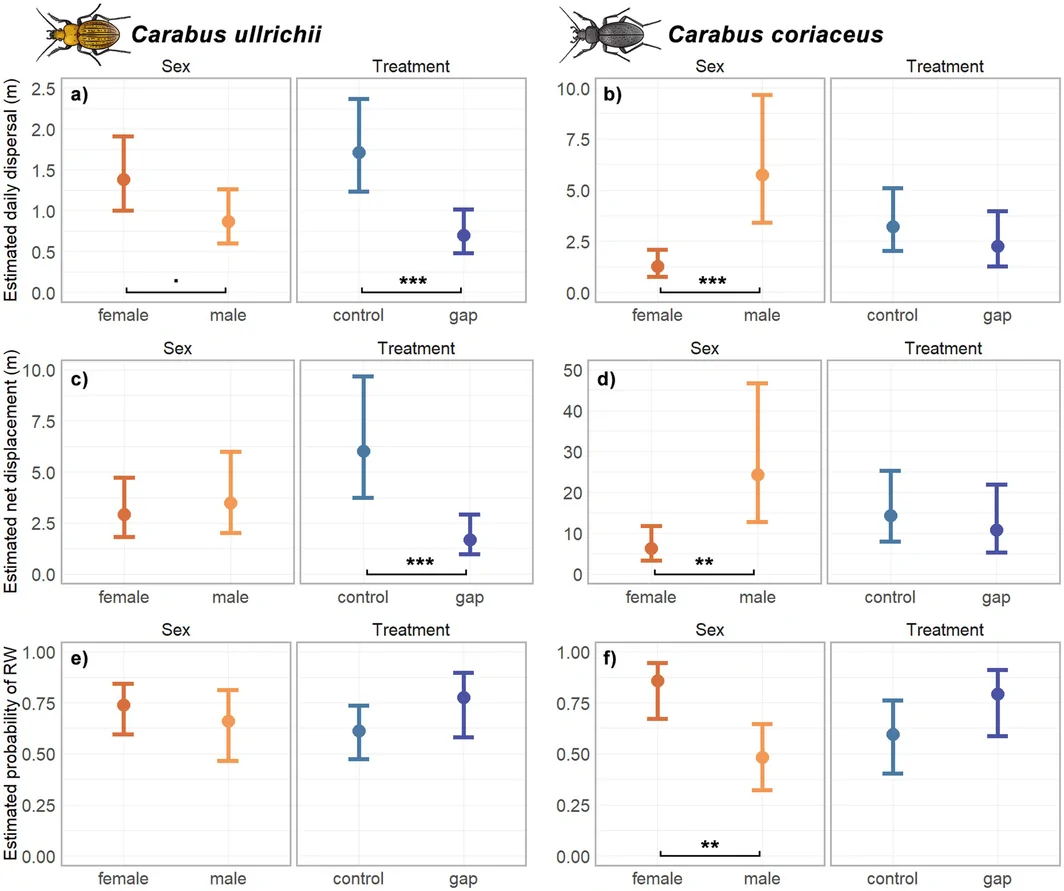
Estimated marginal means of species‐specific movement responses for *Carabus ullrichii* (left) and *C. coriaceus* (right) by sex and treatment. Daily dispersal (a, b) represents the average distance moved within 24 h, and net displacement (c, d) indicates the straight‐line distance between the first and last locations. The probability of random walk (RW; e, f) was derived from hidden Markov models. The full dots indicate the predicted means, and vertical lines represent 95% confidence intervals. Statistical significance is shown as: “.” for marginal trend (*p* < 0.1), “*” for *p* < 0.05, “**” for *p* < 0.01, and “***” for *p* < 0.001.

Finally, species‐specific GLMs indicated that daily dispersal of *C. ullrichii* was only marginally higher in females and significantly greater in the closed‐canopy control forest compared to gaps (Table 2, Figure 3a). The treatment effect was also observed for the net displacement, being higher in the control forest than in gaps (Figure 3c). However, the proportion of random walk in individual trajectories did not differ between sexes or treatments (Figure 3e). In *C. coriaceus* , males showed significantly higher daily dispersal, net displacement, and a lower proportion of random walks than females (Table 2, Figure 3b,d,f), while treatment did not affect either response.

**TABLE 2:** Summary of generalized linear models for species‐specific movement responses by sex and treatment.

Species	Response	Explanatory variable	*χ* ^2^	df	*p*
*C. ullrichii*	Daily dispersal	**Intercept**	**15.812**	**1**	**< 0.001**
		Sex	3.429	1	0.064
		**Treatment**	**12.670**	**1**	**< 0.001**
	Net displacement	**Intercept**	**35.822**	**1**	**< 0.001**
		Sex	0.211	1	0.646
		**Treatment**	**11.901**	**1**	**< 0.001**
	Proportion of RW	Intercept	3.501	1	0.061
		Sex	0.585	1	0.444
		Treatment	2.078	1	0.149
*C. coriaceus*	Daily dispersal	Intercept	1.728	1	0.189
		**Sex**	**17.113**	**1**	**< 0.001**
		Treatment	0.913	1	0.339
	Net displacement	**Intercept**	**25.216**	**1**	**< 0.001**
		**Sex**	**8.616**	**1**	**0.003**
		Treatment	0.354	1	0.552
	Proportion of RW	**Intercept**	**5.658**	**1**	**0.017**
		**Sex**	**8.182**	**1**	**0.004**
		Treatment	2.283	1	0.131

*Note:* Significant effects are highlighted in bold, marginal trends (*p*‐value between 0.05 and 0.1) are underlined.

## 4 Discussion

Using radio telemetry, we found that small‐scale gap cuttings in temperate forest represent a heterogeneous but suitable habitat matrix that is perceived differently by two carabid species. Regarding our first research question on habitat recognition, it seems that only *C. ullrichii* individuals were able to recognize gap cuttings as distinct habitat patches, thereby reducing their daily dispersal and net displacement and keeping within their boundaries, while *C. coriaceus* appeared to be indifferent to gaps. We found no evidence that individuals of either species could distinguish between circular and elongated gaps, which does not support our second research question regarding the sensitivity of carabids to gap geometry. At this small scale, the differences in edge‐to‐interior ratios between the two gap shapes are likely insufficient to create environmental signals that beetles can distinguish. Finally, regarding species specificity, our results highlight contrasting behavioral strategies: while the smaller *C. ullrichii* had more sedentary, area‐limited movement patterns within gap cuttings, the larger *C. coriaceus* showed high individual variability and strong sex‐dependent movement, with males covering greater distances and having a higher tendency for directed movement than females. Taken together, these findings suggest that gap cuttings implemented under CCF may be attractive habitat patches for some large carabids, but do not act as local barriers to the movement of other species.

Focusing on *C. ullrichii*, HMM results indicate that the closed‐canopy forest and gap cuttings are preferred environments. Across both treatments, trajectories were dominated by random walks (60%–80% of active fixes), which is typical for high‐quality habitat patches where individuals exhibit area‐restricted foraging. If the habitats were less suitable, the proportion of random walk would typically fall below 40% as beetles adopt directed movement to escape from the area as quickly as possible (Růžičková and Elek 2023). This high suitability across the study site suggests that gaps implemented under CCF can indeed successfully maintain the characteristics of semi‐natural forest stands. However, while both habitats were suitable, they were used by beetles in fundamentally different ways. In the gaps, we observed lower daily dispersal rates and much lower net displacement, suggesting a pronounced patch fidelity. This behavior is consistent with the high sensitivity of *C. ullrichii* to light intensity, leading to a preference for open forest stands as well as forest edges (Kádár et al. 2017; Růžičková and Veselý 2018). Since the microclimate of gap cuttings offers improved light conditions compared to the relatively uniform interior of a closed‐canopy forest, gaps can effectively mimic these favorable edge conditions (Gálhidy et al. 2006; Schliemann and Bockheim 2011; Horváth et al. 2023). During the spring breeding season of *C. ullrichii* (from late April to June), adults likely prefer gaps because increased soil moisture, resulting from a lack of tree transpiration, and dense vegetation cover provide better sheltering options and a higher biomass of prey (Jordan et al. 1999; Boros et al. 2019). However, overall warmer and drier conditions later in summer can pose severe thermal stress and desiccation risks for developing larvae (Homburg et al. 2019; Růžičková et al. 2025). We also found that females of *C. ullrichii* tended to be more active than males, which corresponds with the early‐season activity peak of females during the reproductive period (Kádár et al. 2017; Růžičková and Veselý 2018). Although both sexes of *C. ullrichii* are capable of covering relatively long distances, reaching up to 20 m in 3 h at the forest edge (Růžičková and Veselý 2018), their movement in gaps was far more localized here based on visual evaluation of trajectories. This suggests that individuals of *C. ullrichii* released in gaps settled into a localized foraging behavior where their needs for heat, moisture, and food were met at once, whereas they exhibited larger‐scale movement in the forest interior.

In contrast, the larger *C. coriaceus* perceived the forest‐gap matrix as a suitable and functionally contiguous landscape without any detectable treatment response. The dominance of random walks in trajectories confirms that both habitats represent a suitable environment for this species, too. With an average step length nearly four times that of *C. ullrichii* and individuals covering distances of up to 181 m, these beetles can cross gaps in a matter of hours. While *C. coriaceus* can also be considered an “abrupt” forest edge specialist (Riecken and Raths 1996), these 300 m^2^ gaps are likely too small to be perceived as distinct patches. Unlike larger clear‐cuttings that significantly alter the movement of *C. coriaceus* (Elek et al. 2021), the small‐scale openings in this study do not appear to reach the threshold necessary to affect its behavior. In addition, as an autumn breeder with mostly biennial development, *C. coriaceus* encounters gaps characterized by high microclimatic variability, including cold, humid autumn mornings and high temperature peaks in the afternoon (Horváth et al. 2023). However, as a more robust beetle (body size: 33–40 mm) that walks mostly on the surface of the forest floor, it is likely better physiologically buffered against these fluctuations than the smaller, litter‐dwelling *C. ullrichii* (body size: 22–33 mm; Hůrka 1996; Thiele 1977). The primary driver for the movement of *C. coriaceus* in our study appeared to be its reproductive strategy, which occurs from late August to November (Turin et al. 2003). The significant sex bias, with males exhibiting much higher daily dispersal, greater net displacement, and a lower probability of random walks than females, points toward active mate‐seeking (Kádár et al. 2015). In addition (although it has not been tested), we observed increased activity of beetles during approaching weather fronts. This suggests that *C. coriaceus* may also respond to fluctuations in atmospheric pressure, similar to other insects as a means of optimizing periods of activity (Thiele 1977; Pellegrino et al. 2013). This suggests that for *C. coriaceus* , the drive to find a mate during the autumn breeding season, perhaps synchronized by atmospheric cues, masks any potential avoidance or attraction to the microclimatic conditions found within the gaps.

Beyond species‐level trends, we observed high individual variability in movement activity, ranging from wide‐ranging “explorers” to sedentary “sluggards.” This phenomenon has also been documented by radio telemetry in other large carabid species (Negro et al. 2017; Bérces and Růžičková 2019; Edwards et al. 2026). While present in both species, this variation was particularly noticeable in *C. coriaceus* . Such strong behavioral variability can potentially bias responses at the species level, suggesting that movement in these carabids is driven not only by the environment or biological traits like sex, but also by inherent, individual‐specific attributes. Simple summary metrics, including daily dispersal and net displacement, provide a necessary measure of movement magnitude, while HMMs are well‐suited to identify the underlying behavioral states, including their transition probabilities and movement structure regardless of high individual variability. Combining these methods allows for a simultaneous evaluation of fine‐scale behavioral changes and actual movement activity in relation to habitat utilization.

Finally, some methodological limitations must be acknowledged, which are common for radio‐tracking studies on large carabids and insects in general (Růžičková and Elek 2026). The short battery life of small transmitters restricts tracking duration to approximately 15 days, preventing full‐season monitoring of individual movement patterns. Furthermore, high equipment costs and unpredictable predation may restrict sample size, particularly for *C. ullrichii* in our case. The high individual variability in behavior combined with the relatively low sample size therefore means that our results represent a case study rather than a generalization across populations of either species.

## 5 Conclusion

Our radio‐tracking study demonstrates that small‐scale gap cuttings implemented under Continuous Cover Forestry can be an effective tool for enhancing forest heterogeneity without disrupting connectivity. We showed that these forestry interventions did not affect overall habitat suitability for either species. However, they may trigger divergent movement responses in ground‐dwelling insects that remain completely undetected by traditional assemblage‐level sampling. In our study, gaps did not act as local barriers to movement; instead, they served as high‐quality patches for the sedentary and more sensitive *C. ullrichii*, while remaining easily traversed by the larger, wide‐ranging *C. coriaceus* . These individual‐level findings complement previous research at broader scales by providing a behavioral mechanism underlying observed species or assemblage changes. Ultimately, the use of gap cuttings as part of Continuous Cover Forestry ensures that managed temperate forests remain both permeable and resource‐rich. This forestry approach supports the coexistence of carabid species with contrasting environmental requirements and dispersal capacities, making it a viable strategy for maintaining biodiversity in the managed temperate forests of Europe.

## Author Contributions

**Jana Růžičková:** conceptualization (equal), data curation (equal), formal analysis (equal), funding acquisition (equal), investigation (equal), validation (equal), visualization (equal), writing – original draft (equal). **Áron Székely:** investigation (equal), writing – review and editing (equal). **Péter Ódor:** conceptualization (equal), funding acquisition (equal), methodology (equal), writing – review and editing (equal). **Zoltán Elek:** conceptualization (equal), data curation (equal), formal analysis (equal), funding acquisition (equal), investigation (equal), methodology (equal), writing – original draft (equal).

## Funding

This study was supported by the Hungarian National Research Development and Innovation Fund (PD 146480, K 143270) and the #oakadapt project of the Interreg VI‐A Hungary‐Slovakia Program (HUSK/2302/1.2/168). It has also received funding from the HUN‐REN Hungarian Research Network.

## Conflicts of Interest

The authors declare no conflicts of interest.

## Supporting information


**Data S1:** Annotated R script for all data processing, statistical analyses, and figures generated in this study.

**Data S2:** Movement data used in this study.

**Figure S1:** Fitted state‐dependent probability densities from the top‐ranking species‐specific hidden Markov models (selected from 1000 candidate models). Histograms show step lengths (a, c) and turning angles (b, d) for *Carabus ullrichii* (top) and *C. coriaceus* (bottom). Curves represent the fitted distributions for the overall pool of the data (dashed black line) and the two behavioral states tested in the models: random walk (orange) and directed movement (blue).
**Figure S2:** Diagnostic plots of DHARMa simulated residuals assessing model fit for generalized linear models of movement responses for *Carabus ullrichii* (left) and *C. coriaceus* (right). Rows represent the three analyzed responses: daily dispersal (a, b), net displacement (c, d), and the proportion of random walk (e, f).

## Data Availability

Raw movement data and the R script used in this study are available as [Link], [Link].
