# Supplementary material for: Context‐Dependent Movement Responses of Large Carabids to Forest Gap Cuttings
Source: Ecol Evol. 2026 Aug 28;16(9):e74250. doi: 10.1002/ece3.74250 (PMC13525330; doi:10.1002/ece3.74250)
Supplement: Supplementary file 1 [file ECE3-16-e74250-s003.docx]

**Online supplementary material**

**Context-dependent movement responses of large carabids to forest gap cuttings**

Jana Růžičková, Áron Székely, Péter Ódor and Zoltán Elek

**Figure S1:** Fitted state-dependent probability densities from the top-ranking species-specific Hidden Markov Models (selected from 1,000 candidate models). Histograms show step lengths (a, c) and turning angles (b, d) for *Carabus ullrichii* (top) and *C. coriaceus* (bottom). Curves represent the fitted distributions for the overall pool of the data (dashed black line) and the two behavioral states tested in the models: random walk (orange) and directed movement (blue).

**Figure S2:** Diagnostic plots of DHARMa simulated residuals assessing model fit for generalized linear models of movement responses for *Carabus ullrichii* (left) and *C. coriaceus* (right). Rows represent the three analyzed responses: daily dispersal (a, b), net displacement (c, d), and the proportion of random walk (e, f).
